# Ligands and Receptors of the Interleukin-1 Family in Immunity and Disease

**DOI:** 10.3389/fimmu.2013.00396

**Published:** 2013-11-20

**Authors:** Cecilia Garlanda, Alberto Mantovani

**Affiliations:** ^1^Department of Inflammation and Immunology, Humanitas Clinical and Research Center, Rozzano, Italy; ^2^Department of Biotechnology and Translational Medicine, University of Milan, Rozzano, Italy

**Keywords:** interleukin-1, decoy receptor, inflammation, innate immunity, adaptive immunity, autoinflammatory diseases

IL-1 has served as a ground breaking molecule in immunology and it is now experiencing a renaissance. Originally the description of a cytokine acting at vanishingly low concentration on cells and organs as diverse as the hypothalamus (fever) and T cells (1) was without precedent in biology and paved the way to the whole field of cytokines and their pleiotropic mode of action.

The discovery of the importance of IL-1 in defense against bacteria and of the Toll-IL-1 resistance (TIR, as originally defined) domain was upstream of the discovery of Toll-like receptors (2). Along the same line, the identification of MyD88 as the key adaptor in the IL-1 receptor signaling cascade (3) prompted its identification in Toll/TLR4 signaling (4, 5). The type II IL-1 receptor was identified as a decoy for IL-1, thus providing a new paradigm in receptor biology (6), subsequently extended to other cytokines and growth factors (7). Stunning from this strong roots, IL-1 has in recent years seen a renaissance. New relatives of IL-1 and IL-1R have been identified and their function has been defined in innate and adaptive immune responses. IL-1 family members have emerged as key players in the differentiation of the main T helper subsets, Th1, Th2, and Th17. Finally, anti-IL-1 strategies have had a tremendous impact in autoinflammatory diseases and are being tested in a variety of clinical conditions.

This volume brings together eight articles that are intended to provide a summary about IL-1 family ligand and receptors in inflammation and immunity. The eight articles are briefly described below.

van de Veerdonk et al. focus their review on the IL-1 family of ligands, describe their biological functions and provide new insights in their biology (8). In particular they focus on the new IL-1 family members, IL-37 and the cytokines belonging to the IL-36 subfamily and on the potency of blocking IL-1 in disease. Among the ligands, a special focus on the biology of IL-18 as well as its role in human disease is provided by the review by Dinarello et al. (9). IL-18 is synthesized as an inactive precursor requiring processing by caspase-1 into an active cytokine, similarly to IL-1β, and is constitutively present in nearly all cell types. The activity of IL-18 is balanced by the presence of a high affinity naturally occurring IL-18 binding protein (IL-18BP), which is now in clinical trials.

Most members of the IL-1 family, including the master pro-inflammatory cytokine IL-1β, are leaderless proteins and are released from the cell though a “non-classical” pathway of secretion. Rubartelli et al. review current hypotheses on the mechanisms of externalization of IL-1 family members and discuss their relevance with respect to the different functions, as cytokines or as DAMPs, played by IL-1 family members (10).

Members of IL-1R like receptor family include signaling molecules and negative regulators. In our review, we present the latter, which include the prototypic decoy receptor type 2 IL-1R and “receptors” with regulatory function, such as TIR8/SIGIRR (11). We suggest that the presence of multiple pathways of negative regulation of members of the IL-1/IL-1R family emphasizes the need for a tight control of members of this fundamental system, which mediates potentially devastating local and systemic inflammatory reactions.

Voronov et al. present the role of IL-1 as a pleiotropic cytokine in the context of cancer (12). In their secreted form, IL-1α and IL-1β are involved in tumorigenesis and tumor invasiveness, whereas IL-1α, when expressed on the cell membrane, stimulates anti-tumor cell immunity. Differential patterns of IL-1α and IL-1β expression and function have been observed in different tumors, thus the authors suggest that better understanding of the role of IL-1α and IL-1β in distinct malignancies will enable the application of novel IL-1 modulation approaches in cancer patients as an adjunct to conventional approaches.

Lopetuso et al. discuss the dichotomous functions of IL-1 family members, such as IL-1, IL-1Ra, IL-18, and IL-33, in gastrointestinal-related inflammatory disorders, depending on the phase of disease or homeostasis and show that IL-37 is emerging as a potent anti-inflammatory cytokine which downregulates colitis (13). In addition, they present data on IL-1 family members suggesting novel pathogenic hypotheses and translational implications for inflammatory bowel disease (IBD) and inflammation-associated colorectal cancer.

The review by Federici et al. presents inherited autoinflammatory diseases secondary to mutations of proteins of the intracellular pathways deputed to the activation and secretion of IL-1β (14). The authors show that the understanding of the molecular pathways involved in these disorders has clarified that similar pathogenic mechanisms play also a crucial role in sustaining inflammation in several multi-factorial inflammatory disorders and opened new perspectives for the treatment of these autoinflammatory disorders based on IL-1 blockers.

Finally, Santarlasci et al. discuss the involvement of IL-1α and IL-1β in the differentiation, activation, and maintenance or survival of the different Th cell subsets (15). Indeed, the differential expression of IL-1R1 on human CD4^+^ T cell subsets confers distinct capacities to acquire specific effector functions. In particular, IL-1β is a key cytokine in Th17 development, acting through IL-1R1 expressed already by the naïve CD4^+^ Th17 precursor, and interestingly by a sub-set of Th1 cells possibly derived by plasticity of Th17 cells.

The reviews collected in this issue of Frontiers will hopefully provide the reader with the sense of diversity and impact of IL-1 family members in the activation and regulation of innate and adaptive immune responses and in immunopathology.
